# Case report: Radiography and computed tomography of tension pneumoperitoneum caused by gastric perforation in a dog

**DOI:** 10.3389/fvets.2023.1281966

**Published:** 2024-01-11

**Authors:** Myounghun Kim, Jeongyun Jeong, Changhyeon Cho, Kidong Eom, Jaehwan Kim

**Affiliations:** Department of Veterinary Medical Imaging, College of Veterinary Medicine, Konkuk University, Seoul, Republic of Korea

**Keywords:** computed tomography, dog, gastric perforation, radiography, tension pneumoperitoneum

## Abstract

Tension pneumoperitoneum is characterized by excessive accumulation of gas in the peritoneal cavity, which leads to cardiorespiratory distress. We present the case of a 4-year-old female Labrador retriever who presented with a severe abdominal distension and panting. Radiography revealed a large volume of free gas in the peritoneal cavity with decreased serosal detail. After emergency needle decompression, ultrasound-guided aspiration of the peritoneal effusion helped confirm septic peritonitis. Computed tomography revealed a gastric mass measuring approximately 3.7 × 5.0 × 5.5 cm, which was suspected to have caused the gastric perforation. A large volume of free gas was present in the peritoneal cavity, causing compression and centralization of the abdominal organs. A low-attenuating cleft suggestive of perforation site near the gastric mass was also observed. Exploratory laparotomy confirmed gastric perforation of approximately 2.2 cm adjacent to the gastric mass. The patient was finally diagnosed with tension pneumoperitoneum caused by gastric perforation. The mass was resected with a 1–2-cm surgical margin, and imprinting cytology indicated gastric carcinoma. The patient was aggressively treated with fluid, analgesic, antithrombotic, and antibacterial therapy. However, the patient’s condition continued to deteriorate, and euthanasia was performed at the owner’s request. Our report is the first to describe the multimodal imaging features of a dog with tension pneumoperitoneum secondary to gastric perforation caused by gastric neoplasm.

## 1 Introduction

Tension pneumoperitoneum (TPP) is a type of pneumoperitoneum characterized by the excessive accumulation of gas in the peritoneal cavity, leading to cardiorespiratory distress (1–5). Excessive gas within the peritoneal cavity increases intraabdominal pressure and pushes the diaphragm cranially, leading to respiratory distress accompanied by tachypnea (1, 2). Excess gas compresses the caudal vena cava and reduces venous return, ultimately leading to hypotension and tachycardia (1, 2). Therefore, urgent decompression is necessary to reduce intraabdominal pressure.

For the prompt diagnosis of TPP, diagnostic imaging plays a pivotal role. Distinctive signs indicative of TPP, including cranial displacement of the diaphragm, medial displacement of the liver, central crowding of viscera, and the presence of massive pneumoperitoneum, can be identified through abdominal radiography (2, 6). To investigate the causes of TPP, Computed tomography can be helpful. The most common causes of TPP, gastrointestinal (GI) perforation, is characterized on CT by the discontinuity of the GI wall at the site of perforation or by accompanying indirect findings suggestive of GI perforation.

## 2 Case description

A 4-year-old intact female Labrador retriever dog, weighing 21 kg, presented with anorexia and lethargy for 20 days. Physical examination revealed severe abdominal distension, fever (40.7°C), increased heart rate (132 bpm) and breathing difficulty accompanied by panting.

Blood examination revealed leukocytosis (35.1 × 10^9^ cells/L; reference range: 6–17 × 10^9^ cells/L) with severe neutrophilia (30.3 × 10^9^ cells/L; reference range: 3.9–8 × 10^9^ cells/L) and monocytosis (2.3 × 10^9^ cells/L; reference range: 0.2–1.1 × 10^9^ cells/L). Plasma biochemical analysis revealed increased levels of alkaline phosphatase (825 U/L; reference range: 29–155 U/L), amylase (1768 U/L; reference range: 388–1,007 U/L), lactate (4.9 mg/dL; reference range: 0.5–2.5 mg/dL), C-reactive protein (59.9 mg/L: reference range: 0–20 mg/L), and D-dimer (2.0 mg/dL; reference range: 0–0.3 mg/dL). The serum glucose level was within the reference range (77 mg/dL; reference range: 67–147 mg/dL).

**Figure 1:**
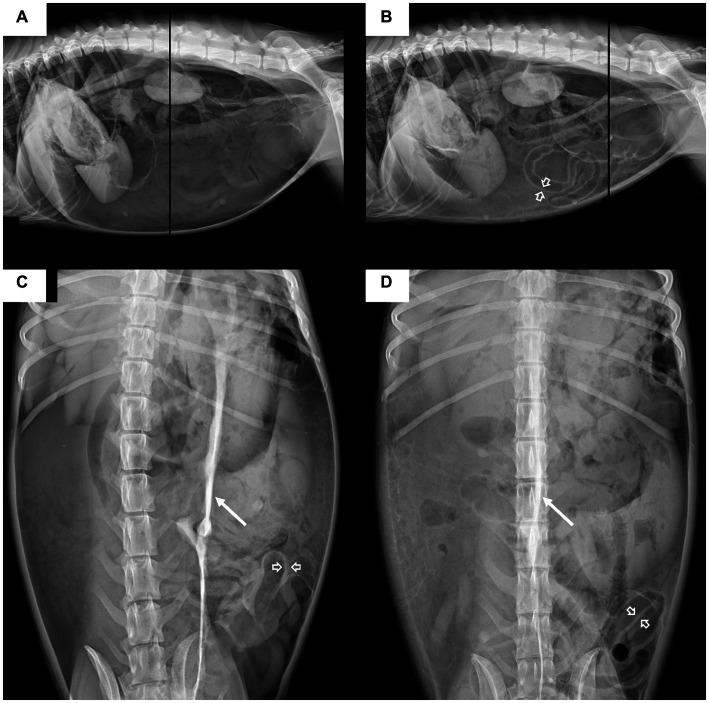
Lateral **(A,B)** and ventrodorsal **(C,D)** abdominal radiographic projections of the dog. The images on the left **(A,C)** were obtained before decompression, and those on the right **(B,D)** were obtained immediately after decompression. Before decompression, massive pneumoperitoneum is characterized by the outlining of the peritoneal cavity with massive amount of gas and outlining of both sides of the small bowel wall with peritoneal gas (open arrow); a decreased overall abdominal serosal detail is also noted. On the ventrodorsal **(C)** radiograph, a radiopaque stripe (arrow) is identified in the mid-abdomen, which is considered a peritoneal ligament. After decompression, the abdominal distension decreased, but a large amount of free gas is still present. Cranial displacement of the diaphragm indicates increased intraperitoneal pressure.

Abdominal radiography (Titan 2000; Comed Medical Systems Co., Ltd., Seoul, Korea; 82 kVp, 10.2 mAs, 320 mA) revealed a large volume of free gas in the peritoneal space (Figures 1A,C) with marked abdominal distension and cranial displacement of the diaphragm. Peritoneal gas was observed outlining the outer wall of the small bowel. In addition, a radiopaque stripe of approximately 7 mm in thickness and 25 cm in length was identified on the mid-abdomen, suggestive of a ligament or peritoneal reflection. The overall abdominal serosal detail was reduced. The radiographic findings indicated that the panting and breathing distress were caused by massive pneumoperitoneum. Emergent needle decompression and peritoneal effusion sampling were performed; however, despite improvement in respiration and reduction in abdominal distension, a large amount of free gas was persistent (Figures 1B,D). In the peritoneal fluid analysis, a significant elevation in white blood cells (> 17 × 10^9^ cells/L; reference range: < 7 × 10^9^ cells/L) and total protein (3.6 g/dL; reference range: < 2.0 g/dL) was observed and the glucose concentration was 28 mg/dL.

**Figure 2:**
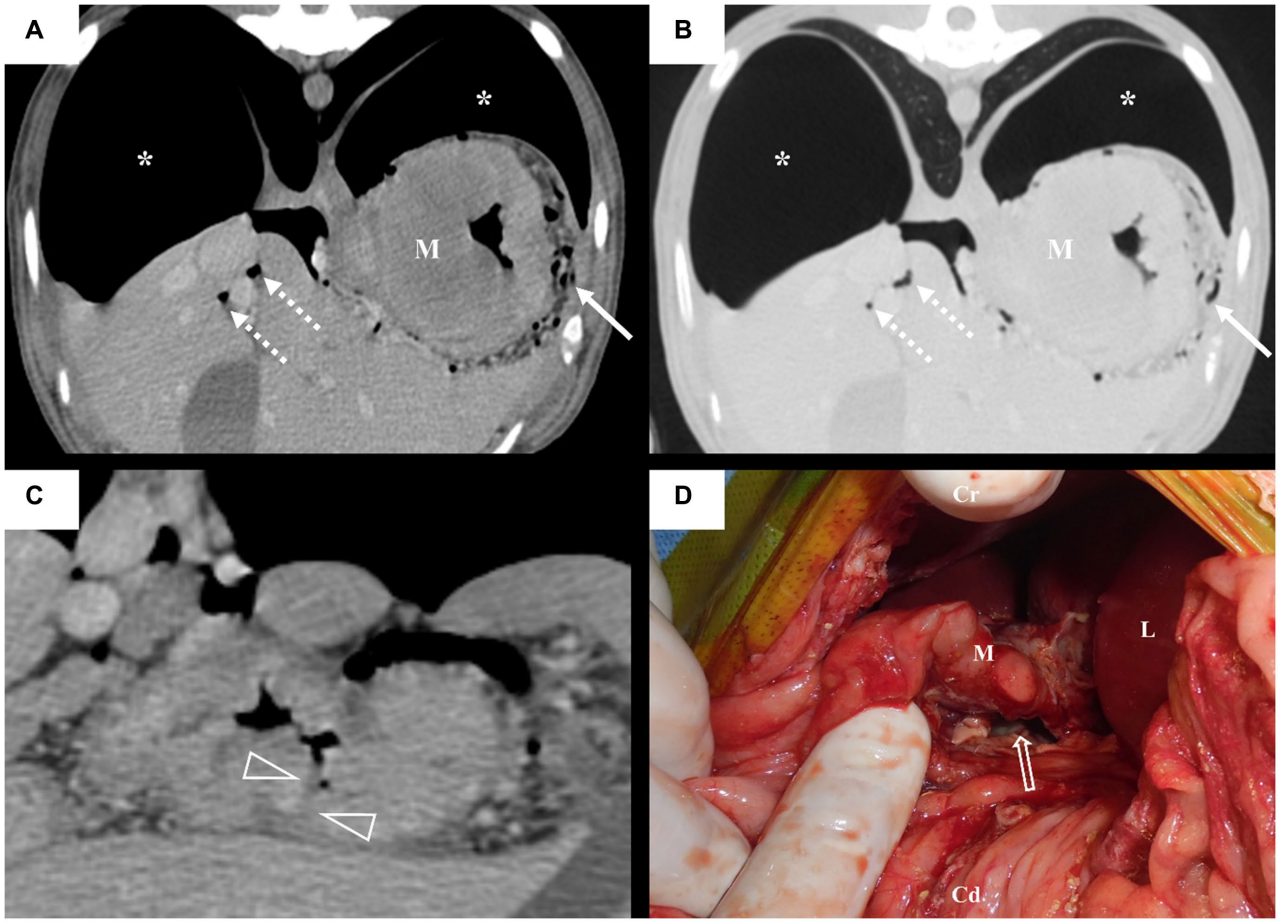
Transverse **(A–C)** multiplanar post contrast computed tomography (CT) images of the patient with a soft tissue algorithm (WL 45, WW 450) **(A,C)** and lung algorithm (WL -400, WW 1500) **(B)**. Intraoperative image **(D)** of the dog. The gastric mass (M) with homogeneous contrast enhancement is seen in the cardia. A low attenuating cleft (arrowhead) adjacent to the gastric mass is presumed to be the perforation site. Notice the gastric mass (M) and perforation site (open arrow) after the separation of the omental adhesion. A large volume of free gas (asterisk) is presence and displaces the liver medially. Note that the structure thought to be the omentum covers the stomach and numerous gas (arrow) present between them. Multiple gas bubbles (dashed arrow) are trapped between peritoneum. **(A,B)** images are on the same level but in different algorithms. CT images, same kv, mAS, and thickness as in the document. L: liver, M: gastric mass, Cr: cranial, Cd: caudal.

**Figure 3:**
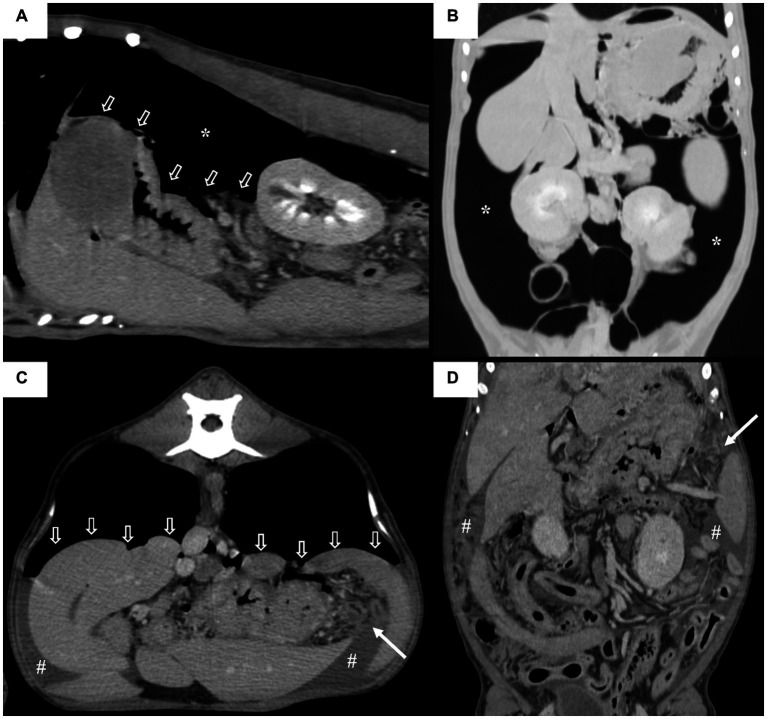
Sagittal **(A)**, dorsal **(B,D)** and transverse multiplanar **(C)** post contrast computed tomography (CT) images of the patient with a soft tissue algorithm (WL 45, WW 450) **(A,C,D)** and lung algorithm (WL -400, WW 1500) **(B)**. Note that a large amount of gas (asterisks) compresses the intra-abdominal organs in the gravity dependent direction (open arrows) and displaces them centrally on the dorsal plane. Diffuse peritoneal effusion (#) and the fat stranding signs are observed throughout the abdominal cavity, with the fat stranding sign being particularly pronounced around the stomach (arrows).

Computed tomography (CT) was performed using a 164-detector row CT scanner (Brivo CT 385; GE Medical Systems, Milwaukee, WI, United States; 120 kVp, 110 mAs, 1.25-mm slice thickness) with the dog under general anesthesia and positioned in ventral recumbency. A power injector (CT Power Injector; GE Medical Systems, Milwaukee, WI, United States) was used to administer 600 mg iodine/kg iohexol (Omnihexol 300; Korea United Pharmaceutical, Seoul, Korea). In post-contrast images, we found a gastric mass with homogeneous enhancement, measuring approximately 3.7 × 5.0 × 5.5 cm and extending from the cardia to the fundus of the stomach (Figure 2). Small gas bubbles were present between the omentum and gastric mass (Figures 2A,B). A substantial amount of free gas was present in the peritoneal cavity (Figure 2), causing compression and centralization of the abdominal organs (Figures 3A–C). Diffuse peritoneal effusion and fat stranding were observed throughout the peritoneal cavity, particularly around the stomach (Figure 3D). Furthermore, a low-attenuating cleft that could be indicative of perforation site was observed near the gastric mass. These imaging findings led to a tentative diagnosis of gastric perforation caused by gastric neoplasia, resulting in TPP and peritonitis.

The dog underwent exploratory laparotomy for identifying and correcting the perforation site and performing peritoneal lavage. A gastric perforation site (Figure 2D) of approximately 2.2 cm was identified near the gastric mass. Severe generalized peritonitis with fibrin deposition along the peritoneum and slightly opaque yellow ascites were observed. The mass was resected with a surgical margin of 1 ~ 2 cm, and after peritoneal lavage and drainage, the abdomen was closed. Biopsy was not performed owing to a lack of the owner’s consent. However, imprinting cytology indicated gastric carcinoma. The patient was treated with fluid therapy, analgesic, antithrombotic agents, and antibacterial therapy. Despite aggressive treatment, the patient’s condition worsened, and euthanasia was performed the day after surgery at the owner’s request.

## 3 Discussion

In this case report, we described a case of TPP in a dog, in which imaging findings led to a tentative diagnosis of gastric perforation caused by gastric neoplasia, TPP, and peritonitis. GI perforation can cause TPP through a “one-way valve” mechanism, in which the liver and omentum covering the perforation site act as a valve, allowing air to escape from the GI tract but preventing it from reentering the tract (1, 2, 7). However, TPP is a rare consequence of GI tract perforation and can occur in conjunction with aerophagia (4). In the present case, aerophagia is likely to have occurred owing to panting caused by abdominal pain, ultimately leading to TPP (8). In addition, the omentum around the perforation site may have acted as a valve, preventing the air from reentering the stomach.

TPP has been reported to have gastrointestinal, pulmonary, and urological causes in humans. Previous studies showed that GI causes (27/36) were the most common (6). To date, three veterinary cases of TPP have been reported. Of them, one case was experimentally induced in a dog, whereas the remaining two occurred in cats and were caused by perforations in the stomach and duodenum (4, 5, 7). Although the number of cases is small, the present and previous cases indicate that GI perforation may be the primary cause of TPP in veterinary medicine, similar to that in humans. To the best of our knowledge, no cases of spontaneous TPP caused by gastric perforation in a dog has been reported. Here, we report a case of TPP caused by gastric perforation in a dog.

Gastric perforation in dogs has several causes, including gastric ulcers, neoplasms, abdominal trauma, and iatrogenic causes. It occurred in approximately 35–45% of dogs with gastroduodenal ulcerative disease and primary neoplasms (9–11). In this case, there was no history of trauma or iatrogenic factors, and the presence of perforation around the gastric mass suggests a diagnosis of spontaneous perforation due to gastric carcinoma. Spontaneous perforation caused by gastric tumor is known to occur when ulceration and tumor necrosis extend to the deep layers of the gastric wall, specifically the subserosa (11). While gastric wall rupture due to gastric cancer is reported to occur in humans at rates ranging from 0.56 to 3.9%, there have been no documented frequency of tumor induced perforation in dogs (12).

The diagnosis of TPP in humans is based on the clinical signs and abdominal radiographic findings (13). The clinical signs of TPP include tympanitic abdominal distension, respiratory distress, and hemodynamic collapse, which are associated with massive pneumoperitoneum. In the present case, breathing distress and tympanitic abdominal distension were observed. Further, the characteristic abdominal radiography findings of TPP in humans include cranial displacement of the diaphragm, saddle bag sign indicative of medial displacement of liver, central crowding of viscera, football sign indicative of massive pneumoperitoneum, where the abdominal cavity is outlined by gas, and Rigler sign indicative of large amounts of pneumoperitoneum when gas is outlining both sides of the bowel wall (2, 6). Additionally, a radiopaque stripe was identified on abdominal radiography. This finding can resemble those observed in humans with a massive pneumoperitoneum, attributed to peritoneal ligaments such as the falciform ligament or umbilical ligaments (13). However, it is important to note that in this case, imaging was performed in the prone position (ventral recumbency), as opposed to humans imaged in the supine position (dorsal recumbency). Therefore, there are anatomical differences in the identity of the radiopaque stripe, and it is considered to be associated with one of the peritoneal ligaments. Although the exact nature of the radiopaque stripe could not be precisely determined, its similarity to features observed in humans with massive pneumoperitoneum suggests that it could be helpful in the diagnosis of TPP (2, 3, 7).

In humans, CT findings of TPP include compression of multiple bowel loops by massive amount of gas, centralization of abdominal organs, and compression of the inferior vena cava. Similar findings were observed in our case, including compression of the small intestine and centralization of the abdominal organs. However, no compression of the caudal vena cava was observed; this may be attributable to the emergency decompression before CT or ventral recumbency during imaging, which differs from the position used during CT in humans. Therefore, the CT findings of TPP may be similar among dogs and humans, indicating that the CT diagnostic criteria used for humans may be applicable to dogs.

In humans, TPP caused by GI tract perforation requires laparotomy and continuous intraperitoneal drainage (2–7), and CT is a commonly used diagnostic tool to detect GI perforations. Discontinuities in the GI wall, which typically appear as low-attenuation clefts on CT, should be identified to accurately locate the perforation. The frequency of detecting these discontinuities is generally less than 50% (14, 15). Despite this low frequency, the predictive value for the site of perforation is relatively high, ranging from 82 to 90%. Indirect findings such as intestinal wall thickening, abnormal wall enhancement, abscess, and inflammatory masses located near the perforation site are also helpful in predicting the perforation site (16). In this case, there was an area around the gastric mass that raised suspicion of a perforation site; however, due to adhesion to the omentum, the presence of ascites, and a collapsed stomach, it was difficult to definitively confirm the perforation site (14). Despite the difficulty in accurately localizing the perforation site on CT, a perforation near the gastric mass was anticipated because of the focal gastric wall thickening (16, 17). From this perspective, CT may help not only to determine the need for laparotomy but also to plan the surgical procedure in patients with TPP.

The diagnosis of septic peritonitis is based on increased white blood cell count, higher total protein levels, and peritoneal fluid glucose concentration. In this case, we observed elevated white blood cells counts (> 17 × 10^9^ cells/L; reference range: 7 × 10^9^ cells/L), increased total protein levels (3.6 g/dL; reference range: <2.0 g/dL), and peritoneal fluid glucose levels consistently below 55 mg/dL (sensitivity 57%, specificity 100%), with a difference of more than 20 mg/dL (sensitivity 100%, specificity 100%) compared to the blood glucose level. This confirms the diagnosis of septic peritonitis (15, 18). Therefore, the abnormalities noted in the blood tests, including severe neutrophilia, monocytosis, increased C-reactive protein, and D-dimer levels, are indicated of severe inflammatory response.

In conclusion, TPP is a rare but life-threatening complication of gastric perforation in dogs. Imaging modalities, such as radiography and CT, are valuable tools for the diagnosis and detection of its underlying causes. In this case, massive pneumoperitoneum was detected on both radiography and CT, with characteristic findings such as gas outlining the peritoneal cavity, gas on both sides of the bowel wall, cranial displacement of the diaphragm, and a radiopaque stripe on abdominal radiographs. CT also revealed intra-abdominal visceral compression and centralization of the abdominal organs. Moreover, CT was particularly useful in diagnosing the underlying cause of TPP in the present case: gastric perforation caused by a gastric tumor.

## Data availability statement

The original contributions presented in the study are included in the article/supplementary material, further inquiries can be directed to the corresponding author.

## Ethics statement

Ethical review and approval were not required for the animal study because the case report is a description of a clinical case. Written informed consent was obtained from the patients/participants for the publication of this case report.

## Author contributions

MK: Conceptualization, Investigation, Writing – original draft, Writing – review & editing. JJ: Visualization, Writing – review & editing. CC: Visualization, Writing – review & editing. KE: Visualization, Writing – review & editing. JK: Conceptualization, Visualization, Writing – review & editing, Writing – original draft.
